# Evaluation of the Antibacterial Effects of Flavonoid Combination from the Leaves of *Dracontomelon dao* by Microcalorimetry and the Quadratic Rotary Combination Design

**DOI:** 10.3389/fphar.2017.00070

**Published:** 2017-02-17

**Authors:** Yang Li, Houlin Xia, Mingquan Wu, Jiabo Wang, Xiaohua Lu, Shizhang Wei, Kun Li, Lifu Wang, Ruilin Wang, Pan Zhao, Yanling Zhao, Xiaohe Xiao

**Affiliations:** ^1^College of Pharmacy, Chengdu University of Traditional Chinese MedicineChengdu, China; ^2^China Military Institute of Chinese Medicine, 302 Military Hospital of ChinaBeijing, China; ^3^Department of Integrative Medical Center, 302 Military Hospital of ChinaBeijing, China; ^4^Clinical Trial Center, 302 Military Hospital of ChinaBeijing, China

**Keywords:** *Dracontomelon dao* (Blanco) Merr. et Rolfe, antibacterial activity, *E. coli*, microcalorimetry, quaternary quadratic general rotary unitized design

## Abstract

Skin infectious disease is a common public health problem due to the emergence of drug-resistant bacteria caused by the antibiotic misuse. *Dracontomelon dao* (Blanco) Merr. et Rolfe, a traditional Chinese medicine, has been used for the treatment of various skin infectious diseases over 1000 of years. Previous reports have demonstrated that the leaves of *D. dao* present favorable antibacterial activity against *Escherichia coli, Pseudomonas aeruginosa, Staphylococcus aureus*, and *Bacillus subtitles*. The flavonoids are the main components of the ethyl acetate extract of *D. dao* leaf. However, the correlation between flavonoids and antibacterial activities is yet to be determined. In this study, the combined antibacterial activities of these flavonoids were investigated. Three samples with the different concentrations of flavonoids (S1–S3) were obtained. By microcalorimetric measurements, the results showed that the IC_50_ value of S2 was lower than those of S1 and S3. The contents of main flavonoids (including Luteolin, L-Epicatechin, Cianidanol, and Quercetin) in S1–S3 were various, confirmed by the method of the Ultra High Performance Liquid Chromatography (UPLC). Based on the method of quadratic general rotary unitized design, the antibacterial effect of single flavonoid, and the potential synergistic effects between Luteolin and Quercetin, Luteolin and Cianidanol were calculated, which were also proved by microcalorimetric analysis. The antibacterial activities of main flavonoids were Luteolin > Cianidanol > Quercetin > L-Epicatechin. Meanwhile, the synergistic effects of Luteolin and Cianidanol (*P*_*L*+*C*_ = 1.425), Quercetin and Luteolin (*P*_*L*+*Q*_ = 1.129) on anti-microbial activity were validated. Finally, we found that the contents of Luteolin, L-Epicatechin, Cianidanol, Quercetin were 1061.00–1061.00, 189.14–262.86, 15,990.33–16,973.62, 6799.67–7662.64 ng·ml^−1^ respectively, with the antibacterial rate over 60.00%. In conclusion, this study could provide reference for quality evaluation and pharmacodynamics research of *D. dao*.

## Introduction

Traditional Chinese medicine has a long history in the treatment of skin infections. The ancient Chinese people treated skin and soft tissue infections (SSTIs) with Kushen (*Sophora flavescens* Ait.) and Baixianpi (*Dictamnus dasycarpus* Turcz) 2000 years ago. *Dracontomelon dao* (Blanco) Merr. et Rolfe belongs to the Anacardiaceae family, a traditional Chinese medicinal material with regional feature, and has been widely used to treat various infectious diseases, such as decubitus and skin ulcers. Previous studies have showed that the ethanol extracts of the leaves of *D. dao* demonstrate anti-*Staphylococcus aureus* and anti-*Bacillus subtitles* activities (Khan and Omoloso, 2002). The subsequent studies from our research team have also indicated that the different extracts from the leaves of *D. dao* by means of systematic solvent extraction (including petroleum ether, chloroform, ethyl acetate, n-butanol, and water) show anti-*Escherichia coli* (the greatest IC_50_ value is 83.93 μg·ml^−1)^ and anti-*Staphylococcus aureus* (the greatest IC_0_ value is 98.5 μg·ml^−1^) activities, especially the ethyl acetate fraction, the main components of which were flavonoids, including Cianidanol, L-Epicatechin, Quercetin, and Luteolin (Liu T. et al., 2013; Zhao et al., 2015), exhibit significant anti-*Pseudomonas aeruginosa* (IC_50_ = 18.06 μg·ml^−1^) activity (Wu et al., 2015). The metabolic power-time (*P-t*) curves were established (Kong et al., 2009b; Braissant et al., 2010; Ren et al., 2010; Kabanova et al., 2012). Moreover, the essential oil from the leaves of *D. dao* has been reported to have anti-tumor activity (Su et al., 2008). Taken together, extracts from the leaves of *D. dao* have possessed a high anti-infectious potential.

Microcalorimetry is a rapid, effective and sensitive technique of biological dection with no invasion and destructiveness to the subjects (Vor et al., 2002; Kong et al., 2009b; Wang et al., 2010), which has been widely used to investigate and evaluate the effect of natural product extracts on the metabolism of bacteria or cells (Baldoni et al., 2009; von et al., 2009; Manneck et al., 2011; Wenzler et al., 2012). The basic principle of microcalorimetry is that the growth or/and metabolism of organism consistent with the change of heat is real-time online recorded and further evaluated. Under a set of growth conditions, unique power—time curves presented by the heat produced in the growth of cells could be analyzed quantitatively and qualitatively by the microcalorimeter (Zhao et al., 2010, 2011; Liu T. et al., 2013; Zheng et al., 2014).

In our present study, three samples with the different concentrations of flavonoids (S1–S3) were obtained, and the combined antibacterial activities of flavonoids from these samples were investigated. The concentrations of these four flavonoids were measured by UPLC, and the parameters were calculated by the quadratic general rotary unitized design to predict the antibacterial effects of single and combined flavonoids with different concentration.

This study is aimed to investigate the main anti-bacterial components of the leaves of *D. dao* and analyze the interaction of flavonoids from leaf extracts, specifically to provide reference for the future research.

## Materials and methods

### Samples, chemicals, and reagents

The leaves of *D. dao* (Blanco) Merr. et Rolfe were purchased from the Chinese herbal medicine market in Guangdong province of China and were authenticated by Professor Xiaohe Xiao (Department of Integrative Medical Center, 302 Military Hospital of China, 100, the 4th Ring Road, Beijing 100039, China.). The leaves were dried in shade and stored at room temperature.

Leaves of *D. dao* were crushed into powder, and decocted with eight times ultra-pure water by refluxing for 1.5 h with the procedure repeated again. After the combined extract filtered and evaporated, the water decoction was further extracted by ethyl acetate. Then ethyl acetate fraction was eluted by 70% alcohol within column chromatography of polyamide and three samples (S1–S3) were collected in succession by removing the solvent.

*Escherichia coli* [ATCC 25922] was provided by the American Type Culture Collection, Manassas, Virginia, USA. *E. coli* was inoculated in Luria-Bertani (LB) culture medium prepared by dissolving 10 g peptone, 5 g yeast extract, and 5 g NaCl in 1,000 ml ultra-pure water (pH 7.0–7.2) and sterilized by autoclaving at 0.1 MPa and 121°C for 30 min and then stored in a refrigerator at 4°C (Zhao et al., 2011). Before microcalorimetric measurements, *E. coli* was added to a 50 ml sterilized flask containing 30 ml LB culture medium and then incubated in incubator shaker for 2.5 h at 37°C (Liu T. et al., 2013) with the rotation speed at 75 rpm.

Polyamide for chromatography (60–100 mesh) was obtained from Mosu (the batch number: 20160223, Shanghai Mosu Science Equipment Co., Ltd., Shanghai, China). Ninety five percent ethanol was from Lircon (the batch number: 151111A, ShanDong LIRCON Medical Technology Co., Ltd., Shandong, China). Pure water was from Wahaha (the batch number: 201601185200TW, Hangzhou Wahaha Group Co., Ltd., Zhejiang, China). Chromatographic grade methanol was from Sigma Chemicals (the batch number: WXBC2019V, Sigma Scientific Co., L.L.C, USA).

The information of reference substance: Quercetin (prepared by laboratory, the degree of purity ≥98%), L-Epicatechin (the batch number: B-020-140923, the degree of purity ≥98%), Cianidanol (the batch number: E-011-140728, the degree of purity ≥98%), Luteolin (the batch number: M-007-150730, the degree of purity ≥98%). All reference substances but Quercetin were purchased from Chengdu Herbpurify bio-technology Co. Ltd., Chengdu, China.

### Instruments

A microcalorimeter of type TAM 3114/3236 Bio-activity monitor (TA, Sweden) was used to determine the metabolic power–time (*P–t*) curves of *E. coli* growth. The TAM air microcalorimeter is an eight-channel heat conduction calorimeter for flow measurements under isothermal conditions. This microcalorimeter was thermostated at 37°C with a temperature error of ±0.02°C. The baseline stability was lower than 40 μW over 24 h (Kong et al., 2015). For more details of the performance and construction of the instrument, see the instruction and the report by Xie et al. (1988).

UPLC were performed using a Waters Acquity UPLC™ system (Waters, Milford, MA, USA), including binary solvent delivery pump, auto sampler manager, column compartment, and photo diode array detector, connected to Waters Empower 2 software.

### Microcalorimetric measurements

#### Sample preparation

50.0 mg powder of flavonoids from *D. dao* was dissolved in 10 mL of LB culture medium. After filtration through millipore filter (pore size: 0.45 μm), sample solution was conducted at the concentration of 5.0 mg/mL for microcalorimetric measurements.

#### Experimental procedure

This experiment was performed using the ampoule method and the microcalorimeter was thermostated at 37°C. One 20 mL sterilized glass ampoule was filled with 10 mL LB culture medium. while others were filled with 0.5 mL LB culture medium containing *E. coli* at a cell density of 1 × 10^6^ colony forming units (CFU)/ml (Kong et al., 2011) and different weight of flavonoids were added into each ampoule with a final volume of 10 ml. Eventually, each ampoule was sealed up and put into the eight-channel calorimeter block. When the temperature of ampoules reached 37°C, the *P-t* curves were recorded at an interval of 1 s until the recorder returned to the baseline. All data were collected continuously using the dedicated software package.

### UPLC analysis

#### Preparation of reference standard solution

The standard solutions were prepared by accurately weighing. 20.0 mg of Cianidanol, L-Epicatechin, Quercetin, or Luteolin was added respectively into 10 mL of methanol to prepare the solutions with a concentration of 2 mg/ml, and then filtered through millipore filter with the pore size of 0.22 μm.

#### UPLC conditions

The chromatographic separation was performed using a Waters Acquity BEH C_18_ column (50 × 2.1 mm, 1.7 μm) with Column temperature of 30°C. The mobile phase composed of solvent A (Chromatographic grade methanol) and solvent B [water solution of 0.15% (V/V) formic acid] was performed with a linear gradient: 0–3.8 min (5%, A), 3.8–4 min (5–30%, A), 4–14.8 min (30%, A), 14.8–15 min (30–50%, A), 15–18.8 min (50%, A), 18.8–19 min (50–95%, A), 19–20 min (95%, A) at a rate of 0.2 mL/min. The detection wavelength was set at 280, 350, 360 nm and the sample injection volume was designated as 2.0 μl, based on the Chinese Pharmacopoeia 2015 edition.

### Statistical analysis: principal component analysis (PCA)

PCA is a famous technique used for reducing the dimensionality of the data, which can visualize the information of the dataset in a few principal components retaining the maximum possible variation within that set (Yi L. Z. et al., 2007; Yi Z. B. et al., 2007; Chen J. et al., 2008; Chen Y. et al., 2008). Hence, the metabolic *P-t* curves were analyzed to obtain the key thermodynamic parameters which reduced the dimensionality by PCA statistics (Kong et al., 2009a; Liu S. X. et al., 2013). The SPSS statistics software (SPSS for Windows 17.0, SPSS) was used during this process.

### Quaternary quadratic general rotary unitized design

There are many restrictions on the orthogonal test, for instance, the multiple test numbers should be considered and the contribution of factors to the index is always uncertain and uncontrollable. Although the uniform design test could save test duration, the accuracy of results cannot be guaranteed. The statistical model of quaternary quadratic general rotary unitized design could not only solve the problems above, but also minimize the shortcomings that the variance of the prediction value of quadratic regression heavily depends on the position of the experimental point in factor space and the interference in error. The quaternary quadratic general rotary unitized design is effective and useful to get a better combination because of its high precision and less test times.

## Results

### Microcalorimetry

#### Normal power–time curves of *E. coli*

**Figure 1:**
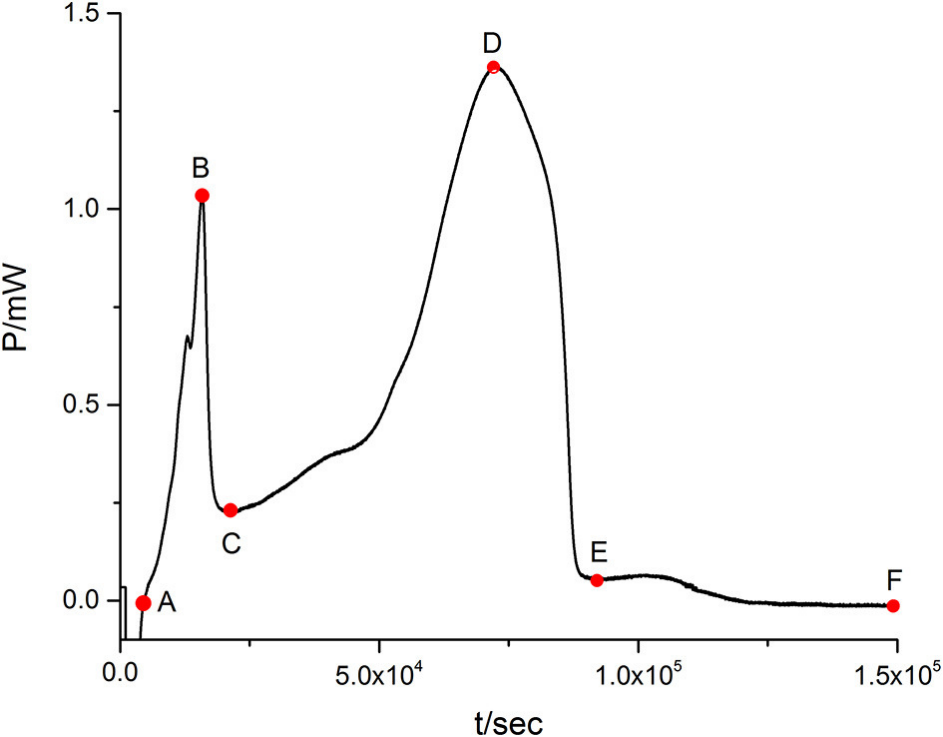
**The normal metabolic ***P-t*** curve of ***E. coli*****. There is a show of five phases: the lag phase (from point A to point B in Figure 1), the first exponential growth phase (from B to C), the transition phase (from C to D), the second exponential growth phase (from D to E), and the decline phase (from E to F).

When the suspensions of *E. coli* are introduced into the ampoules with LB culture medium, the heat-output signals are recorded to form the normal metabolic *P-t* curves of *E. coli* (Figure 1). The heat of bacteria represents its metabolic strength. The curve in Figure 1 shows the typical growth characteristics of *E. coli* and could be divided into five phases: the lag phase (from point A to point B in Figure 1), the first exponential growth phase (from B to C), the transition phase (from C to D), the second exponential growth phase (from D to E), and the decline phase (from E to F).

#### *P-t* curve and quantitative thermo-kinetic parameters for *E. coli* growth with flavonoids

**Figure 2:**
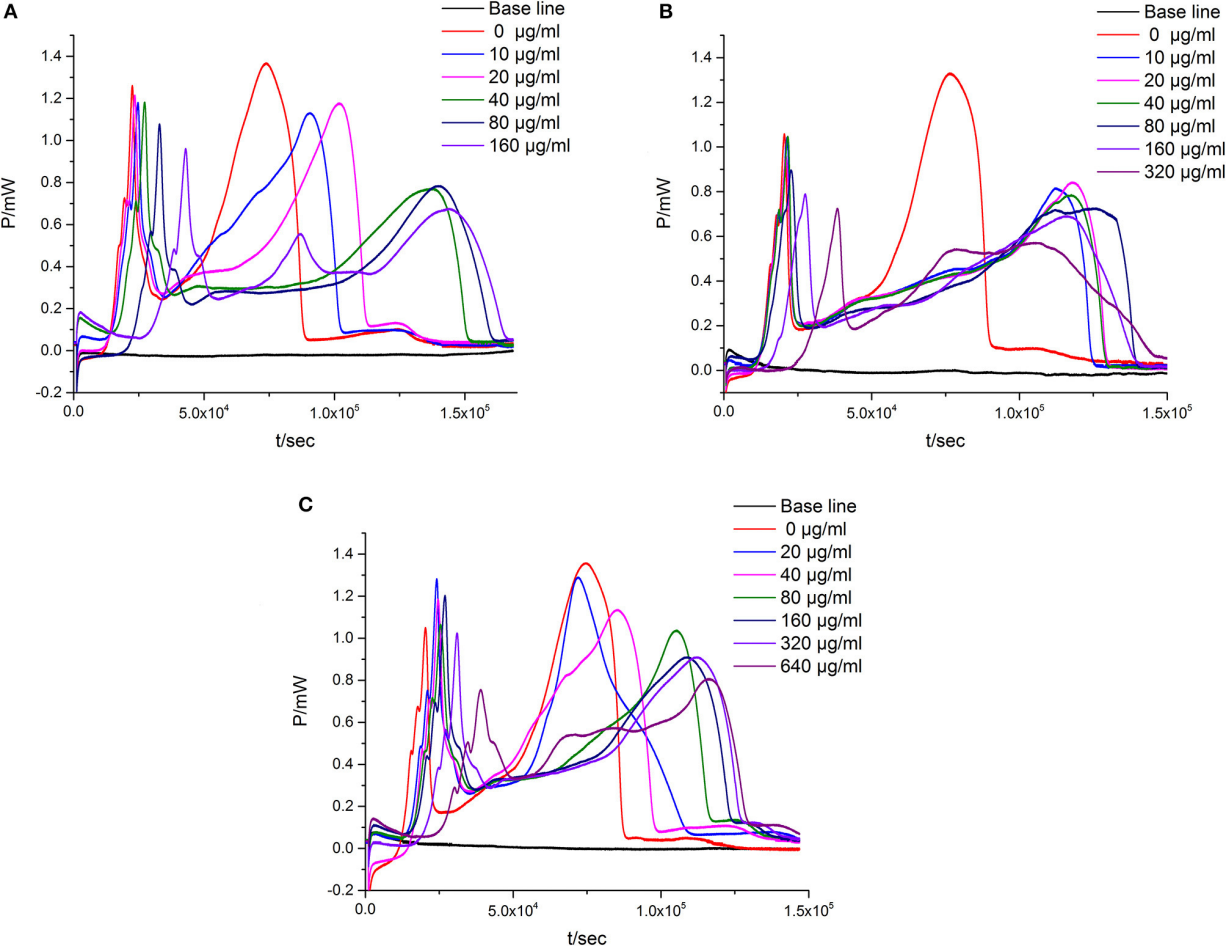
**The ***P-t*** curves of ***E. coli*** intervened by three simples from leaves of ***Dracontomelon dao***. (A)** S1 simple, **(B)** S2 simple, **(C)** S3 simple.

When the suspensions of *E. coli* are introduced into the ampoules with different concentrations of flavonoids, there exists corresponding changes in the *P-t* curve. As could be seen in Figure 2, Table 1, the *P-t* curves of low concentration flavonoids on *E. coli* had slight changes compared with control group. The *P-t* curves and quantitative thermo-kinetic parameters had obvious changes in high concentration, such as the peak of the second exponential growth phase reducing, the time of peak postponing, the slope of curves diminishing and the area of curves lowering. Besides, curves and parameters changed with the concentration increasing. All these demonstrated that the growth of *E. coli* was influenced by flavonoids in different degrees.

**Table 1:** **The thermo-kinetic parameters from the ***P-t*** curves of the growth of ***E. coli*** in the presence of flavonoids from leaves of ***Dracontomelon dao*****.

**Extracts**	**c/μg·ml^−1^**	**t_1_/s**	**Q_1_/J**	**P_1_/mW**	**k_1_/(× 10^−2^s^−1^)**	**t_2_/s**	**Q_2_/J**	**P_2_/mW**	**k_2_ (× 10^−2^s^−1^)**	**I (%)**
S1	0	22,522	10.0200	1.2615	0.03425	73,667	45.3553	1.3683	0.00855	
	10	23,092	11.5878	1.2537	0.03205	102,310	48.5997	1.1886	0.00565	33.92
	20	24,632	10.2405	1.1810	0.02795	90,628	49.5601	1.1322	0.00555	35.09
	40	27,161	10.9591	1.1822	0.02885	137,974	48.9063	0.7740	0.00480	43.86
	80	32,936	12.7095	1.0793	0.02910	140,701	49.4886	0.7855	0.00440	48.54
	160	42,911	9.6241	0.9617	0.01875	143,835	47.1508	0.6767	0.00365	57.31
S2	0	20,457	6.4648	1.0590	0.03025	76,491	44.7235	1.3320	0.00805	
	10	21,340	7.5010	1.0439	0.02425	111,993	43.5841	0.8165	0.00465	42.24
	20	20,980	6.3465	0.9599	0.02040	117,811	47.2773	0.8433	0.00435	45.96
	40	21,576	6.8688	1.0478	0.02545	117,431	45.6901	0.7863	0.00410	49.07
	80	22,659	7.5191	0.8978	0.01520	124,767	49.6248	0.7267	0.00375	53.42
	160	27,459	6.5875	0.7904	0.01135	116,080	44.4874	0.6928	0.00295	63.35
	320	38,276	6.2892	0.7255	0.01040	104,720	41.6386	0.5726	0.00260	67.70
S3	0	20,341	6.1062	1.0509	0.03225	74,366	41.7777	1.3582	0.00860	
	20	24,138	11.4400	1.2826	0.03360	71,819	45.7033	1.2904	0.00595	34.25
	40	24,566	9.9069	1.1863	0.03400	85,225	46.5730	1.1367	0.00530	41.44
	80	25,403	11.2702	1.0661	0.02595	105,095	45.5491	1.0387	0.00515	43.09
	160	26,954	11.8433	1.2042	0.03045	108,463	47.6739	0.9104	0.00455	49.72
	320	30,992	10.4037	1.0251	0.02415	111,791	46.9600	0.9104	0.00410	54.70
	640	38,924	12.6014	0.7557	0.01560	115,887	45.2981	0.8086	0.00370	59.12

#### Dose—response relationship among thermo-kinetic parameters

The influence of flavonoids on the growth of *E. coli* could be quantitatively reflected from the changes of eight thermo-kinetic parameters in Figure 2. The *P-t* curve of *E. coli* growth could be fitted by two exponential functions. They both obey the following equation.

$$\begin{array}{l} P_{t}=P_{0}\times e^{kt}\\ \ln\;P_{t}=\ln\;P_{0}+kt \end{array}$$

In this equation, *P*_0_ and *P*_*t*_ are the power at time 0 and *t*. Using this equation, the quantitative thermo-kinetic parameters such as the power of the first and second peak (P_1_ and P_2_) represent the strength of *E. Coli* metabolism.

The growth rate constants (k_1_ and k_2_) of the first and second exponential phase for *E. coli* growth at 37°C represent the rate of *E. coli* metabolism which is always a thermo-kinetic parameter. The heat outputs in stage 1 and stage 2 (Q_1_ and Q_2_) are obtained from the *P-t* curve of *E. coli* growth affected by different concentrations of flavonoids. The growth rate constant k_1_ and k_2_ and the correlation coefficient *R* (not listed, *R* > 0.9) are also calculated.

### Principal component analysis (PCA) of the eight thermo-kinetic parameters

Eight thermo-kinetic parameters (P_1_, P_2_, t_1_, t_2_, Q_1_, Q_2_, k_1_, and k_2_) which profile the metabolism of *E. Coli* were obtained from the curves (Figure 2). Obviously, these parameters changes were in line with the concentration of flavonoids. It is necessary to find out the main parameter(s) playing crucial role in evaluating the anti-bacterial effects, but the key information reflected from these changes is hard to figure out. Hopefully, PCA could solve these problems well.

The eight quantitative parameters (P_1_, P_2_, t_1_, t_2_, Q_1_, Q_2_, k_1_, k_2_) were analyzed by PCA with the results indicating that the two principal components (Z_*S*1−1_ and Z _*S*1−2_, Z_*S*2−1_, and Z _*S*2−2_, Z_*S*3−1_, and Z _*S*3−2_) contained 90.284, 85.777, and 93.387% information of the original parameters in each sample.

A component correlation matrix of the principal components (**Table 9**) was obtained by PCA analysis. The component correlation of each factor is equivalent to the coefficient of the factor (normalized) in the principal component equation. The equation of main component was formed according to this matrix. Each quantitative parameter is normalized in this equation.

$$\begin{array}{l} {\text{Z}}_{S1-1}=-\;0.913{\text{B}}_{\text{t}1}-0.082{\text{B}}_{\text{Q}1}+0.932{\text{B}}_{\text{P}1}+0.880{\text{B}}_{\text{k}1}\\ -\;0.924{\text{B}}_{\text{t}2}-0.364{\text{B}}_{\text{Q}2}+0.961{\text{B}}_{\text{P}2}+0.942{\text{B}}_{\text{k}2}\\ {\text{Z}}_{S1-2}=-\;0.337{\text{B}}_{\text{t}1}+0.906{\text{B}}_{\text{Q}1}+0.266{\text{B}}_{\text{P}1}+0.404{\text{B}}_{\text{k}1}\\ +\;0.222\text{B}{\text{t}}_{t2}+0.806{\text{B}}_{\text{Q}2}-0.110{\text{B}}_{\text{P}2}-0.248{\text{B}}_{\text{k}2}\\ {\text{Z}}_{S2-1}=-0.804{\text{B}}_{\text{t}1}+0.123{\text{B}}_{\text{Q}1}+0.907{\text{B}}_{\text{P}1}+0.956{\text{B}}_{\text{k}1}\\ -\;0.572{\text{B}}_{\text{t}2}+0.220{\text{B}}_{\text{Q}2}+0.930{\text{B}}_{\text{P}2}+0.949{\text{B}}_{\text{k}2}\\ {\text{Z}}_{S2-2}=-\;0.540{\text{B}}_{\text{t}1}+0.773{\text{B}}_{\text{Q}1}+0.276{\text{B}}_{\text{P}1}+0.000{\text{B}}_{\text{k}1}\\ +\;0.783\text{B}{\text{t}}_{\text{t}2}+0.776{\text{B}}_{\text{Q}2}-0.283{\text{B}}_{\text{P}2}-0.253{\text{B}}_{\text{k}2}\\ {\text{Z}}_{S3-1}=-\;0.931{\text{B}}_{\text{t}1}-0.784{\text{B}}_{\text{Q}1}+0.610{\text{B}}_{\text{P}1}+0.851{\text{B}}_{\text{k}1}\\ -\;0.927\text{B}{\text{t}}_{\text{t}2}-0.584{\text{B}}_{\text{Q}2}+0.971{\text{B}}_{\text{P}2}+0.933{\text{B}}_{\text{k}2}\\ {\text{Z}}_{S3-2}=-\;0.235{\text{B}}_{\text{t}1}+0.451{\text{B}}_{\text{Q}1}+0.786{\text{B}}_{\text{P}1}+0.504{\text{B}}_{\text{k}1}\\ -\;0.052{\text{B}}_{\text{t}2}+0.794{\text{B}}_{\text{Q}2}-0.047{\text{B}}_{\text{P}2}-0.334{\text{B}}_{\text{k}2} \end{array}$$

$\bar{x}$ was the arithmetic mean of this quantitative parameter with *s*_*i*_ the standard deviation in standardized formula.

$$\begin{array}{l} B_{xi}=\frac{x_{i}-\bar{x}}{s_{i}} \end{array}$$

The equation indicated that P_2_, k_1_, and k_2_ might be the main parameters playing more important role in evaluating the anti-bacterial effects of *D. dao* leaf extracts.

### Inhibition ratio *I* of samples S1–S3 on *E. coli*

According to the result of PCA, P_2_, k_1_, k_2_, and Q_2_ might be the main parameters. By further comparison of k_1_ and k_2_ from the equation, we found that k_2_ contributed more than k_1_ to PCA. So, the growth inhibition ratio *I* (%) could be calculated on the basis of k_2_ and can be defined as:

$$I=(\frac{{\text{k}}_{0}-{\text{k}}_{c}}{{\text{k}}_{0}})\times 100$$

Where k_0_ refers to the growth rate constant of the control, k_*c*_ refers to the growth rate constant in the second exponential growth phase at an inhibitor concentration of *c*.

The Probit regression with SPSS statistical analysis software was conducted to calculate the half-inhibitory concentration (IC_50_) after antibacterial rate counted. IC_50_ representing the sensitivity of bacteria to flavonoids was one of the most important indexes in evaluating the anti-bacterial activity of flavonoids. Table 2 showed the 95% confidence limit of IC_50_ for S1–S3.

**Table 2:** **Half inhibition rate of S1–S3 (95% confidence limits)**.

**Extracts**	**IC_50_/μg·ml^−1^**	**Lower limit/μg·ml^−1^**	**Upper limit/μg·ml^−1^**
S1	82.30	49.52	224.29
S2	34.90	16.44	58.94
S3	172.52	97.55	389.56

### UPLC analysis

The UPLC method in this research was newly established with main reference to Chinese Pharmacopoeia 2015 edition and the guidelines of the State Food and Drug Administration of China. We carried out the methodology validation of the UPLC method by Quercetin. External standard calibration lines were generated by injection of standard solutions at five concentrations in triplicate, plotting the peak area (y) obtained from the UPLC analysis against the concentration (x) of the standard material displaying a linear regression with correlation coefficient (r) calculated out.

The results of methodology validation showed that accuracy was obtained by 6 detection resulting in relative standard deviation (RSD) value < 0.99%. Repeatability was determined by analyzing six independent prepared solutions which resulted in RSD value < 1.32%, suggesting that this method had high repeatability. Stability was measured by repeating the analysis of the same sample solution at 0, 3, 6, 12, 24, and 48 h at room temperature with RSD value < 0.96%.

As shown in Table 3, the calibration curves displayed strong linearity over the concentration range of 1–100 μg·ml^−1^ (*r* = 0.9987) for Luteolin, 20–100 μg·ml^−1^ (*r* = 0.9999) for L-Epicatechin, 100–2000 μg·ml^−1^ (*r* = 0.9996) for Cianidanol, 20–800 μg·ml^−1^ (*r* = 0.9999) for Quercetin.

**Table 3:** **the standard curve of four flavonoids**.

**Compound**	**Linear equation**	***r***	**Linear range/μg·ml^−1^**
Luteolin	y = 5764.1x + 490.61	0.9987	1–100
L-Epicatechin	y = 6580.6x – 15,750	0.9999	20–100
Cianidanol	y = 533.64x + 771.56	0.9996	100–2,000
Quercetin	y = 8256.2x – 38,045	0.9999	20–800

The content of Luteolin, L-Epicatechin, Cianidanol, and Quercetin in S1–S3 were determined by external standard method. The linear equations of each standard substance were established through the peak area of five concentration gradient (Table 4).

**Table 4:** **The content of four flavonoids for S1–S3**.

**Compound**	**Extracts**	**Peak Area**	**c/μg·ml^−1^**	**Content/μg·mg^−1^**	**m (%)**
Luteolin	S1	23,419.5	3.978	0.796	0.08
	S2	518,226	89.821	17.964	1.80
	S3	48,179.5	8.274	1.655	0.17
L-Epicatechin	S1	13,183.5	4.580	0.916	0.09
	S2	181,231.5	30.116	6.023	0.60
	S3	128,665.5	22.128	4.426	0.44
Cianidanol	S1	76,331	141.593	28.319	2.83
	S2	793,332	1485.197	297.039	29.70
	S3	903,159	1691.004	338.201	33.82
Quercetin	S1	318,699.5	35.090	7.018	0.70
	S2	5,529,802	705.561	141.112	14.11
	S3	3,093,364	392.084	78.417	7.84

**Figure 3:**
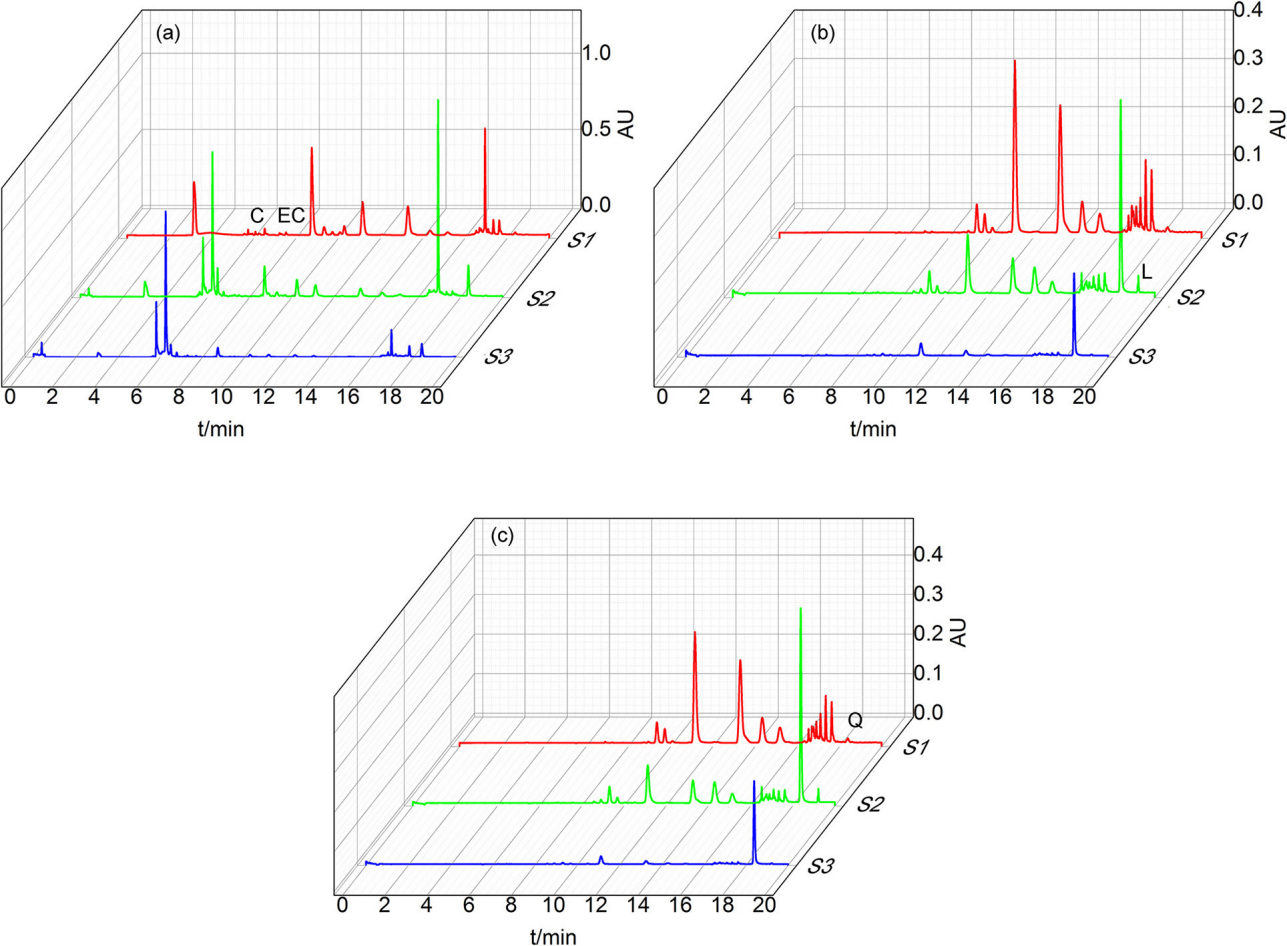
**Chromatograms of S1–S3 under different wavelength. (a)** is shown that the chromatograms under 280 nm in order to detect the content of L-Epicatechin and Cianidanol. **(b)** is shown that the chromatograms under 350 nm in order to detect the content of Luteolin. **(c)** is shown that the chromatograms under 350 nm in order to detect the content of Quercetin. Four peaks were identified by comparison with substances: Cianidanol (C), L-Epicatechin (EC), Luteolin (L), Quercetin (Q).

Figure 3 presented the UPLC characteristic spectrum of S1–S3 from *D. dao* under the optimized condition. The peak of flavonoids was characterized by large areas and good segregation from consecutive peaks.

Figure 3a displayed the spectrum of S1–S3 under 280 nm with the retention time of L-Epicatechin (EC) and Cianidanol (C) 6.8 and 6.0 min, respectively. The content of L-Epicatechin in S1 and S3 were greater than that of S2 with 6.023 μg/mg in S1 and 4.426 μg/mg in S3, 0.60 and 0.44% in total mass, respectively. Likewise, the content of Cianidanol in S2 and S3 was greater than that of S1 with S2 297.039 and S3 338.20 μg/mg which were respectively 29.7 and 33.82% in total mass. Interestingly, the Cianidanol was most abundant among the four flavonoids.

As shown in Figure 3b, the spectrum of S1–S3 under 350 nm exhibited that the retention time of Luteolin (L) was 19.2 min and the content of Luteolin in S2 was much greater than both S1 and S3 with its concentration 89.821 μg/mg accounting for 1.80% in total mass. The spectrum of S1–S3 under 360 nm demonstrated that the retention time of Quercetin (Q) was 18.4 min with its concentration for S2 705.561 μg/mg (accounting for 14.11% in total mass), greater than S1 and S3. While that of Quercetin in S3 was 392.084 μg/mg, holding 7.84% in total mass (Figure 3c).

The total flavonoids for S1-S3 were 37.048, 462.139, and 422.698 μg·mg^−1^ respectively from the UPLC result, suggesting that the content of Luteolin in S2 was far greater than that of Luteolin of S1 and S3. The content of L-Epicatechin and Cianidanol in S2 and S3 was similar, but greater than S1. As for Quercetin, S2 was 2-folds than S3 with both S2 and S3 much >S1.

### Results of quadratic general rotary unitized design analysis

#### Determination of code level

**Figure 4:**
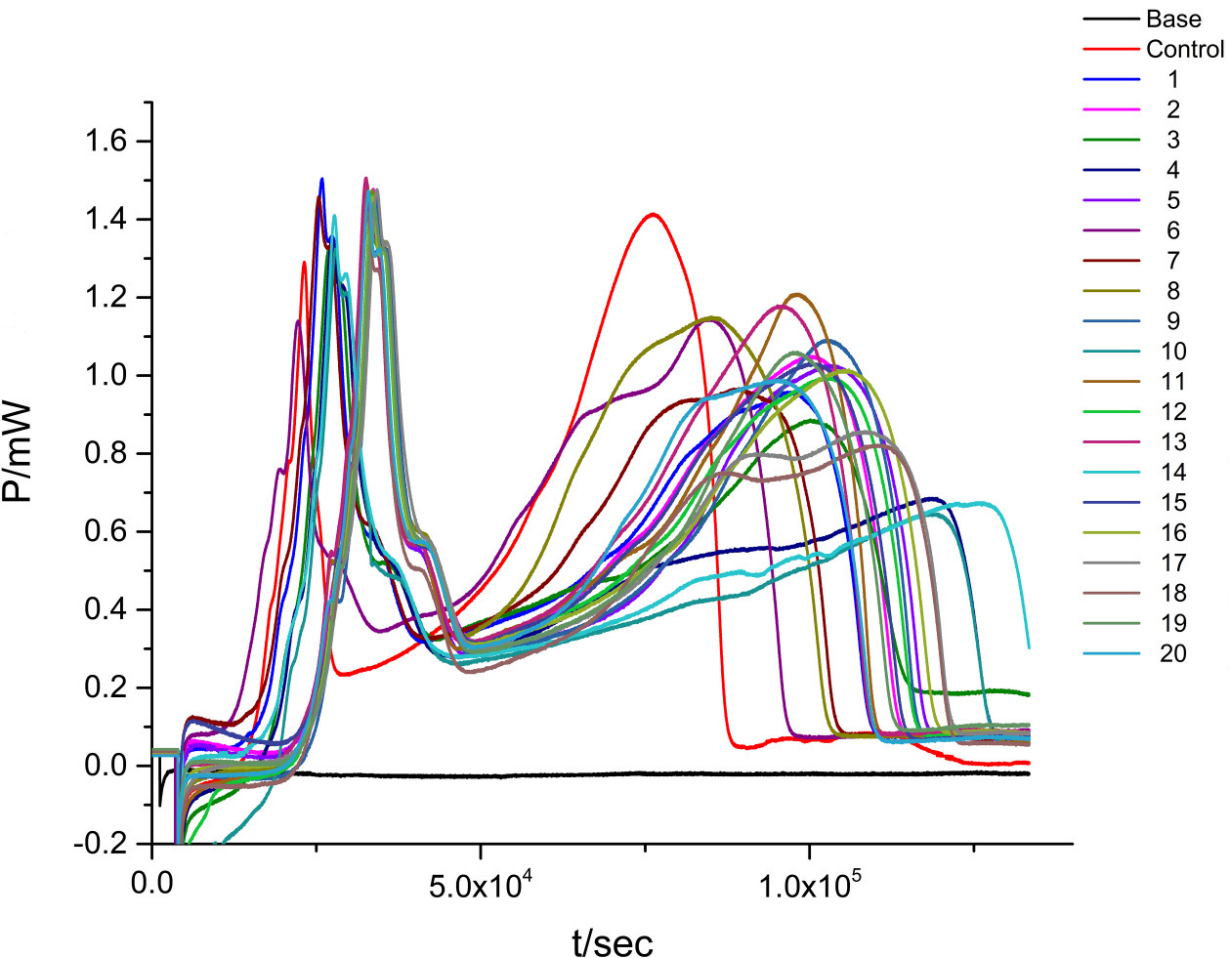
**The ***P-t*** curves of ***E. coli*** at quaternary quadratic general rotary unitized design**.

Based on the IC_50_ of sample, S2 exhibited favorable anti-bacterial effect. The content of Luteolin, L-Epicatechin, Cianidanol, and Quercetin was defined as 0-code, and Luteolin (X_1_), L-Epicatechin (X_2_), Cianidanol (X_3_), and Quercetin (X_4_) were chosen as the four factor. The general quaternary quadratic designs of rotary combination statistical model (1/2 implement) was introduced to investigate the combined effect of four flavonoids with the use of microcalorimeter figuring out the antibacterial rate (Y-value) on different code level. The content of four flavonoids on different code level was illustrated on Table 5 with Table 6 and Figure 4 displaying experimental design scheme and the analysis result in Table 7.

**Table 5:** **The content of four flavonoids on different code level**.

**Code**	**Luteolin /ng·ml^−1^**	**L-Epicatechin /ng·ml^−1^**	**Cianidanol /ng·ml^−1^**	**Quercetin /ng·ml^−1^**
1.6818	1061	354	17,506	8,317
1	906	302	14,947	7,101
0	678	226	11,194	5,318
−1	451	150	7441	3,535
−1.682	296	99	4882	2,319

**Table 6:** **Quaternary quadratic general rotary unitized design**.

**No**.	**X_1_**	**X_2_**	**X_3_**	**X_4_**	**Y (%)**
1	1	1	1	1	49.69
2	1	1	−1	−1	41.51
3	1	−1	1	−1	47.80
4	1	−1	−1	1	44.03
5	−1	1	1	−1	35.85
6	−1	1	−1	1	35.72
7	−1	−1	1	1	40.25
8	−1	−1	−1	−1	27.04
9	−1.6818	0	0	0	34.59
10	1.6818	0	0	0	55.97
11	0	−1.6818	0	0	31.45
12	0	1.6818	0	0	46.54
13	0	0	−1.6818	0	30.19
14	0	0	1.6818	0	47.80
15	0	0	0	−1.6818	33.96
16	0	0	0	1.6818	44.65
17	0	0	0	0	37.11
18	0	0	0	0	40.88
19	0	0	0	0	35.22
20	0	0	0	0	37.11

*X_1_, Luteolin; X_2_, L-Epicatechin; X_3_, Cianidanol; X_4_, Quercetin; Y, antibacterial rate*.

**Table 7:** **The analysis of variance table for quadratic general rotary unitized design**.

**Variable**	***SS***	***f***	***MS***	***r***	***F*****-value**	***p*****-value**
X_1_	293.8219	1	293.8219	0.9103	24.1644	0.0012
X_2_	38.5630	1	38.5630	0.6230	3.1715	0.1128
X_3_	137.9669	1	137.9669	0.8331	11.3466	0.0098
X_4_	57.5721	1	57.5721	0.6974	4.7348	0.0612
${\text{X}}_{1}^{2}$	48.0861	1	48.0861	0.6646	3.9547	0.0819
${\text{X}}_{2}^{2}$	0.0938	1	0.0938	0.0392	0.0077	0.9322
${\text{X}}_{3}^{2}$	0.0938	1	0.0938	0.0392	0.0077	0.9322
${\text{X}}_{4}^{2}$	0.4008	1	0.4008	0.0809	0.0330	0.8604
X_1_X_2_	1.8834	1	1.8834	−0.1733	0.1549	0.7042
X_1_X_3_	0.1509	1	0.1509	−0.0498	0.0124	0.9140
X_1_X_4_	5.8726	1	5.8726	−0.2968	0.4830	0.5068
Linear correlation between X_2_X_3_ and X_1_X_4_						
Linear correlation between X_2_X_4_ and X_1_X_3_						
Linear correlation between X_3_X_4_ and X_1_X_2_						
Regression	934.8898	11	84.9900	*F*_2_ = 6.98,973		0.0021
Residual term	97.2741	8	12.1593			
Lack of fit	80.3727	5	16.0745	*F*_1_ = 2.85323		0.0907
Error	16.9014	3	5.6338			
Total	1032.1639	19				

*X_1_, Luteolin; X_2_, L-Epicatechin; X_3_, Cianidanol; X_4_, Quercetin*.

#### Results of statistical analysis

The regression equations of antibacterial rate were obtained from statistical model of quaternary quadratic general rotary unitized design. That was

$$\begin{array}{l} \text{Y}=37.99545+5.86714{\text{X}}_{1}+2.12554{\text{X}}_{2}+4.02043{\text{X}}_{3}\\ +2.59711{\text{X}}_{4}+2.32473{\text{X}}_{1}^{2}+0.10265{\text{X}}_{2}^{2}+0.10265{\text{X}}_{3}^{2}\\ +0.21225{\text{X}}_{4}^{2}-0.61375{\text{X}}_{1}{\text{X}}_{2}-\;0.17375{\text{X}}_{1}{\text{X}}_{3}-1.08375{\text{X}}_{1}{\text{X}}_{4} \end{array}$$

The result (Table 7) of variance analysis with this model showed that *F*_1_ = 2.85323 (testing of lack of fit for regression equation of antibacterial rate) was < *F*_0.05_(5, 3) = 9.01, which suggested that the X factor had little influence on experimental result. Moreover, the test result for significance was that *F*_2_ = 6.98973 > *F*_0.01_(11, 8) = 5.74, demonstrating that the regression equation had significant difference. The mathematic model with good fitting degree could reflect the experimental file from the two aspects. Hence, the parameters calculated from the model had favorable confidence level.

In single-factor analysis procedure, the *P*-values of X_1_ (Luteolin) and X_3_ (Cianidanol) were < 0.01. Surprisingly, the *P*-values of (L-Epicatechin) and X_4_ (Quercetin) were >0.05. Moreover, the simplified four quaternary regression equation was calculated out without no-significance items of α = 0.10. That was

$$\begin{array}{l} \text{Y}=37.99545+5.86714{\text{X}}_{1}+4.02043{\text{X}}_{3}+2.59711{\text{X}}_{4}\\ +\;2.32473{\text{X}}_{1}^{2} \end{array}$$

The four flavonoids had independent anti-bacterial effect with Luteolin and Cianidanol showing the main and better anti-bacterial effect from the analysis of variance table. In addition, the *P*-value of X_1_ (Luteolin) was less than X_3_ (Cianidanol) and the concentration range of X_1_ was 296–1061 ng·ml^−1^ far less than that of X_3_ (4482–17,506 ng·ml^−1^).

The diagram of single factor effect for S1–S3 was rationally calculated from the rotational combination design model. The single factor under different code level was predicted to make out the antibacterial rate (Y-value) with others factors as 0-code in this method.

**Figure 5:**
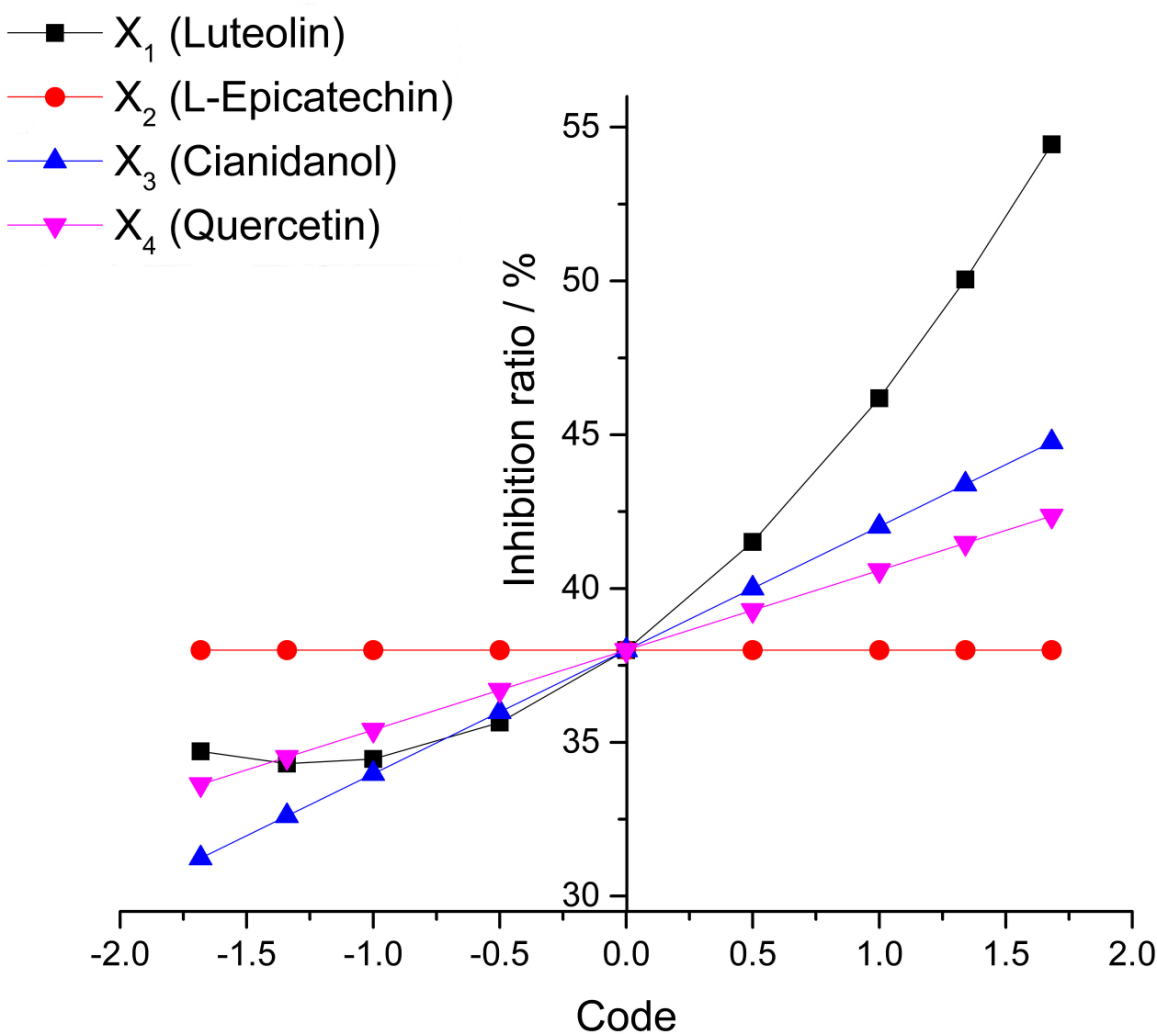
**The analysis of single factor effect diagram**.

The effect of different factors on bacteria could be observed via the range of each factor (the difference of Y-value for the highest code between the lowest one) and change tend single factor in the analysis of single factor effect. The result showed that X_1_ (Luteolin) mainly contributed to the antibacterial rate (Y-value) with X_3_ (Cianidanol) and X_4_ (Quercetin) contributing only marginally. Unexpectedly, X_2_ (L-Epicatechin) had little influence on antibacterial rate. Based on the range result, the sequence of antimicrobial activity was X_1_ (Luteolin) > X_3_ (Cianidanol) > X_4_ (Quercetin) > X_2_ (L-Epicatechin) consistent with the *P*-value of analysis of variance model, further validating the different anti-bacteria effect among the four individual compounds (Figure 5).

**Figure 6:**
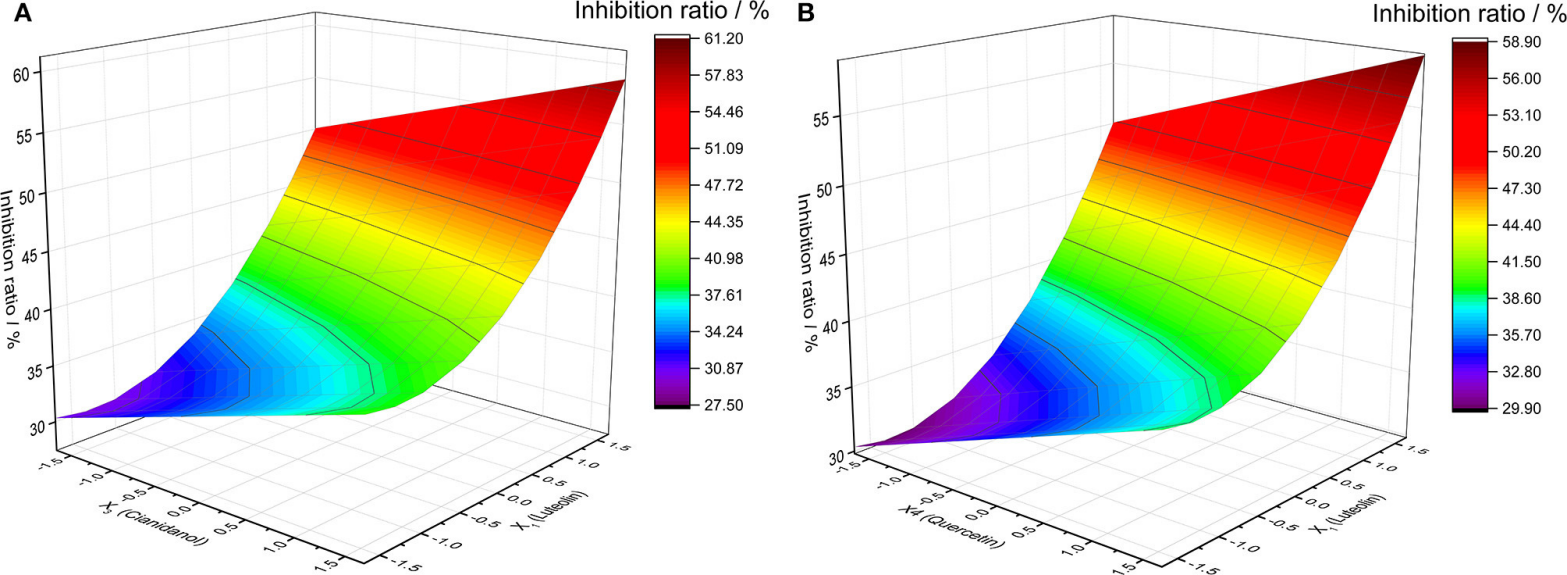
**The analysis of two factors interaction effect diagram. (A)** Luteolin and Cianidanol, **(B)** Luteolin and Quercetin.

Analysis of the effect of two-factor interaction that defined other factor as 0-code for the four flavonoids was obtained from the statistical model of quaternary quadratic general rotary unitized design. The changes of different factors on Y-value as well as the relationship between two factors could be found out via Y-value of two factors under different levels of code by mathematical model. The results showed that there were two couple of factors obvious and similar, such as the pair of Quercetin and Luteolin between Cianidanol and Luteolin (Figure 6).

The diagram of two-factor interaction effect for X_1_ (Luteolin) and X_3_ (Cianidanol) was rationally calculated from the rotational combination design model. When X_1_ and X_3_ factor were in high code level, the synergistic activity was obvious between X_1_ and X_3_ with the antibacterial rate (Y-value) in the top level, which suggested that there was a positive effect between high concentration of X_1_ (Luteolin) and X_3_ (Cianidanol) and antibacterial rate (Y-value). With the decline of code of Luteolin and Cianidanol, Y-value (antibacterial rate) decreased rapidly with a smaller degree. When the X_1_ code number was −1 to −1.6820, the Y-value decreased at first and increased later. When the X_1_ code number was −1.3410 and the X_3_ code number was −1.6818, the Y-value was 27.5466, which is the lowest point in this analysis model. Therefore, we predicted that there existed positive and synergistic effect between Luteolin and Cianidanol. When Luteolin and Cianidanol was in a higher concentration, there existed obvious synergistic effect between them. But when Luteolin and Cianidanol in low concentration, no marked synergistic effect was observed, even presenting slight antagonistic effects similar with the relationship of X_1_ (Luteolin) and X_4_ (Quercetin).

Obviously, the analysis showed that there existed both single factor effect and interaction among two factors. So it was difficult to find out the best antibacterial concentration and combinations from the results of the single factor effect and interaction analysis, and quaternary quadratic regression mathematical model did not have the maximum value of bacterial inhibition rate. The analysis method of frequency domain was performed to analyze and confirm this regression model and the combined optimum concentration (Table 8).

**Table 8:** **Distribution frequency and interval of factor**.

**Code**	**X**_**1**_		**X**_**2**_		**X**_**3**_		**X**_**4**_	
	***n*****_1_**	***f*****_1_**	***n*****_2_**	***f*****_2_**	***n*****_3_**	***f*****_3_**	***n*****_4_**	***f*****_4_**
−1.6818	25	0.0769	65	0.2000	35	0.1077	45	0.1385
−1.0000	25	0.0769	65	0.2000	40	0.1231	45	0.1385
0.0000	50	0.1538	65	0.2000	60	0.1846	65	0.2000
1.0000	100	0.3077	65	0.2000	85	0.2615	85	0.2615
1.6818	125	0.3846	65	0.2000	105	0.3231	85	0.2615
Weight mean	0.7480		0.0000		0.5010		0.3300	
Standard Error	0.0590		0.0690		0.0650		0.0660	
95% confidence limits	0.633–0.864		−0.135–0.135		0.374–0.627		0.200–0.460	
c/ng·ml^−1^	822.32–874.99		215.74–236.26		12,597.62–13,547.13		5674.60–6138.18	

*X_1_, Luteolin; X_2_, L-Epicatechin; X_3_, Cianidanol; X_4_, Quercetin*.

Table 8 showed the frequency distribution of 325 cases with antibacterial rate exceeding 39.87%. when antibacterial rate was over 39.87%, most cases could be obtained, verifying the mathematical regression model. Code value of factors was that X_1_: 0.633–0.864, X_2_: −0.135–0.135, X_3_: 0.374–0.627, X_4_: 0.200–0.460 under the condition that the antibacterial rate >39.87 within 95% confidence limits. In other words, Luteolin was 822.32–874.99 ng·ml^−1^, L-Epicatechin was 215.74–236.26 ng·ml^−1^, Cianidanol was 12,597.62–13,547.13 ng·ml^−1^, and Quercetin was 5674.6–6138.18 ng·ml^−1^.

To minimize the error for this experiment, the combined range was defined as Luteolin 850 ng·ml^−1^ (Code = 0.754), L-Epicatechin 220 ng·ml^−1^(Code = −0.079), Cianidanol 13,000 ng·ml^−1^ (Code = 0.481), and Quercetin 6,000 ng·ml^−1^ (Code = 0.382). The actual antibacterial rate was 45.96% according to the best combination close to the predicted value (46.22%) which further verified the rationality of regression model.

Finally, with the use of validated mathematical model, we finally obtained the combination that Luteolin was 1,061.00–1,061.00 ng·ml^−1^, L-Epicatechin was 189.14–262.86 ng·ml^−1^, Cianidanol was 15,990.33–16,973.62 ng·ml^−1^, Quercetin was 6,799.67–7662.64 ng·ml^−1^ with Y-value (inhibition rate) >60.00 within 95% confidence limits.

### Anti-microbial activity of single flavonoid and combination of two flavonoids

#### Anti-microbial activity of single flavonoid

**Figure 7:**
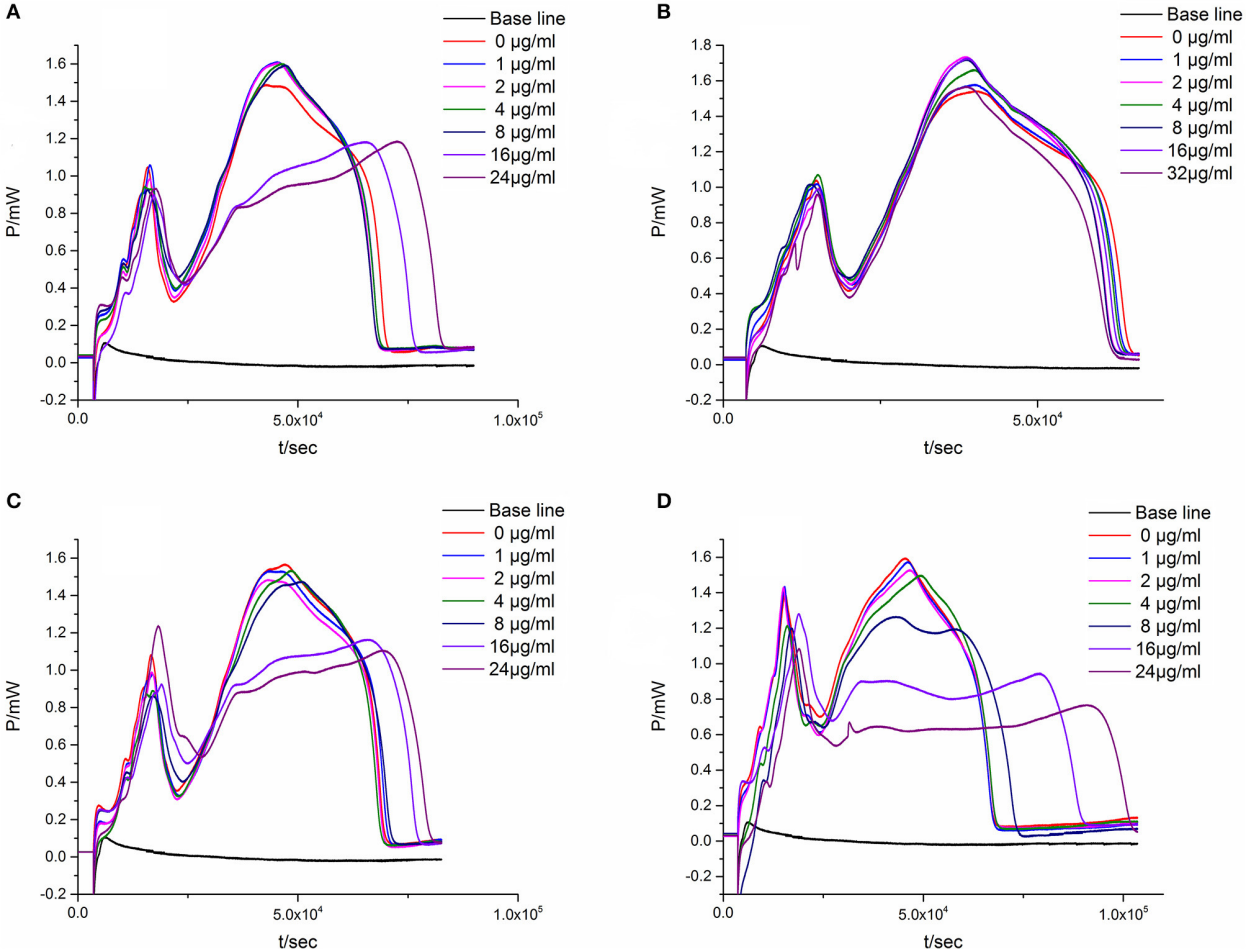
**The ***P-t*** curves of ***E. coli*** intervened by single flavonoid. (A)** Luteolin, **(B)** L-Epicatechin, **(C)** Cianidanol, **(D)** Quercetin.

To verify the result that antibacterial activity of the four major components from the leaves of *D. dao* is Luteolin > Cianidanol > Quercetin > L-Epicatechin based on the quadratic general rotary unitized design model, we determined the anti-bacterial activity of the four flavonoid independently using the microcalorimetry method (Figure 7) with the same parameter as above. The Probit regression with SPSS statistical analysis software was conducted to calculate the half-inhibitory concentration (IC_50_) after antibacterial rate counted. IC_50_ representing the sensitivity of bacteria to flavonoids was one of the most important indexes in evaluating the anti-bacterial activity of flavonoids. Table 9 showed the 95% confidence limit of IC_50_ for each flavonoid. The result showed that Luteolin (15.642 μg·ml^−1^), Cianidanol (17.584 μg·ml^−1^), and Quercetin (19.002 μg·ml^−1^) had lower IC_50_ values, except for L-Epicatechin. When the concentration of L-Epicatechin was increased to 32 μg/ml, the inhibition rate of L-Epicatechin was still < 50%. In the final calculation, only Probit regression can be used to predict the IC_50_ of L-Epicatechin (44.247 μg·ml^−1^). The exact IC_50_ of L-Epicatechin remains to be further analyzed yet, but that the antibacterial activity of L-Epicatechin is much weaker than that of Luteolin, and Cianidanol and Quercetin can be confirmed. By comparing the IC_50_ values, we can confirm that antibacterial activity of the four major components from the leaves of *D. dao* (Luteolin > Cianidanol > Quercetin > L-Epicatechin) based on the quadratic general rotary unitized design model is correct.

**Table 9:** **Component correlation Matrix**.

**Parameters**	**Component**					
	**Z_*S*1−1_**	**Z_*S*1−2_**	**Z_*S*2−1_**	**Z_*S*2−2_**	**Z_*S*3−1_**	**Z_*S*3−2_**
t_1_	−0.913	−0.337	−0.804	−0.540	−0.931	−0.235
Q_1_	−0.082	0.906	0.123	0.773	−0.784	0.451
P_1_	0.932	0.266	0.907	0.276	0.610	0.786
k_1_	0.880	0.404	0.956	0.000	0.851	0.504
t_2_	−0.924	0.222	−0.572	0.783	−0.927	−0.052
Q_2_	−0.364	0.806	0.220	0.776	−0.584	0.794
P_2_	0.961	−0.110	0.930	−0.283	0.971	−0.047
k_2_	0.942	−0.248	0.949	−0.253	0.933	−0.334

#### Anti-microbial activity of the combination of two flavonoids

**Figure 8:**
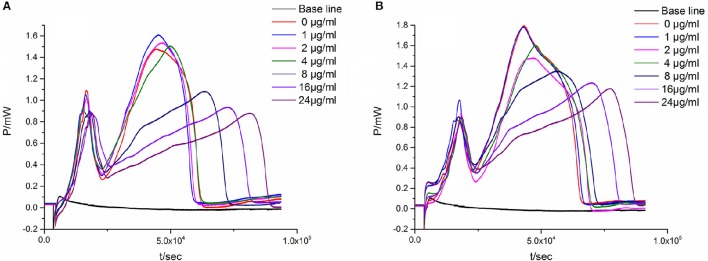
**The ***P-t*** curves of ***E. coli*** intervened by the combination of two flavonoids. (A)** Luteolin and Cianidanol, **(B)** Luteolin, and Quercetin.

In the quadratic general rotary unitized design model, we analyzed two factors interaction effect: Luteolin and Cianidanol, Quercetin, and Luteolin had synergistic effects on antibacterial activity at high doses. In order to verify this result, we independently measured the antimicrobial activity of the two combinations. We referred to the proportion of flavonoids the anti-bacterial rate > 39.87 within 95% confidence limits from the quadratic general rotary unitized design model to determine the ratio of Luteolin and Cianidanol was 1:15, and the ratio of Luteolin and Quercetin was 1:7. microcalorimetry determination of half inhibition rate was also conducted to determine the anti-bacterial activity of flavonoids with the same anti-microbial activity indicators as above (Figure 8). The Probit regression with SPSS statistical analysis software was conducted to calculate the half-inhibitory concentration (IC_50_) after antibacterial rate counted. IC_50_ representing the sensitivity of bacteria to flavonoids was one of the most important indexes in evaluating the anti-bacterial activity of flavonoids. Table 9 showed the 95% confidence limit of IC_50_ for Luteolin and Cianidanol, Quercetin and Luteolin. The result showed that the IC_50_ values of the combination of Luteolin and Cianidanol was 11.566 μg/ml (722.9 ng·ml^−1^ for Luteolin, 10,843.1 ng·ml^−1^ for Cianidanol), and that of the combination of Luteolin and Quercetin was 14.721 μg·ml^−1^ (1840.1 ng·ml^−1^ for Luteolin, 12,880.9 ng·ml^−1^ for Cianidanol) within 95% confidence limits. The ratio of their anti-microbial activity P (in IC_50_ value) through the concentration ratio weighted average method based on the IC_50_ values of the four flavonoids alone and two combinations of two flavonoids was calculated. The results displayed that the IC_50_ value for the combination of Luteolin and Cianidanol was increased to 1.425-fold than the theoretical antibacterial activity of the same concentration of the compound alone and the IC_50_ value for the combination of Luteolin and Quercetin was 1.129-fold as much as the theoretical antibacterial activity of the same concentration of the compound alone. The synergistic effect of Luteolin and Cianidanol (*P*_*L*+*C*_ = 1.425), Quercetin and Luteolin (P_*L*+*Q*_ = 1.129) on anti-microbial activity could be confirmed, since the *P*-values of which were >1.

$$\begin{array}{l} P_{L+C}=\frac{IC_{50\left(L\right)}*\frac{1}{16}+IC_{50\left(C\right)}*\frac{15}{16}}{IC_{50\left(L+C\right)}}\\ P_{L+Q}=\frac{IC_{50\left(L\right)}*\frac{1}{8}+IC_{50\left(Q\right)}*\frac{7}{8}}{IC_{50\left(L+Q\right)}} \end{array}$$

Cianidanol (*C*), Luteolin (*L*), Quercetin (*Q*).

## Discussion

*Escherichia coli*, a Gram negative bacteria, is common and important to screen flavonoids with anti-bacterial activity as a model organism (Sondi and Salopek-Sondi, 2004). In the study, we investigated the anti-*E. coli* effect of three samples from the leaves of *D dao* (Blanco) Merr. et Rolfe by microcalorimetry and UPLC, and demonstrated that the anti-bacterial activity of the three samples (S1–S3) was various after *E. coli* was treated with three samples independently via microcalorimetry. According to the *P-t* curves of *E. coli* in Figure 2 and thermal-kinetic parameters in Table 1, it could be seen that the anti-bacterial activities of different simples varied in a dose-dependent manner with the IC_50_ value sequence S1>S3>S2.

The previous study has demonstrated that flavonoids are abundant in the leaves of *D. dao*, and their anti-bacterial activities have been reported by cumulative evidences. Ethanol extract from *Mentha longifolia* containing the components of Luteolin, Apigenin, Quercetin, and Kaempferol was the most active fraction against the tested bacteria (Akroum et al., 2009). The combination of Luteolin and Amoxicillin displayed synergistic activity against *E. coli* (Eumkeb et al., 2012). Furthermore, the novel Luteolin derivatives showed favorable antibacterial activity *in vitro* against *B. subtilis, S. aureus, P. fluorescens*, and *E. coli* (Lv et al., 2009).

In terms of IC_50_ values, the IC_50_ value of S2 was lower than those of S1 and S3, because the content and ratio of Luteolin, L-Epicatechin, Cianidanol, and Quercetin in S1-S3 were quite different. To be specific, the content of Luteolin in S2 was 22.5- and 10.8-fold than that of S1 and S3, respectively. The content of Quercetin in S2 was 20.1- and 1.8-fold than that of S1 and S3, respectively. However, the content of flavonoids in extracts did not show a linear relationship with the IC_50_ value, indicating that there existed unknown compounds with significant antibacterial activity from the leaves of *D. dao*.

The statistical model of quadratic general rotary unitized design was conducted to analyze the compounds interaction based on the content of four flavonoids in S2. We observed that the *p*-value of Luteolin (*p* = 0.0012) and Cianidanol (*p* = 0.0098) were < 0.01, which indicated that Luteolin and Cianidanol were very important factors (Table 7). Further research is needed to explore their antibacterial mechanism. Of note, Quercetin (*p* = 0.0612) and L-Epicatechin (*p* = 0.1128) were not the major factors of the antibacterial activity in the extract of the leaves of *D. dao*. The ultima result showed that the antibacterial activity of four flavonoids was Luteolin > Cianidanol > Quercetin > L-Epicatechin from the result of single factor analysis. And the IC_50_ values of each flavonoid evaluated by microcalorimetry confirmed the results above (the IC_50_ value of Luteolin was 15.642 μg·ml^−1^; the IC_50_ value of Cianidanol was 17.584 μg·ml^−1^; the IC_50_ value of Quercetin was 19.002 μg·ml^−1^; the IC_50_ value of L-Epicatechin was 44.247 μg·ml^−1^; Table 10).

**Table 10:** **Half inhibition rate of flavonoids (95% confidence limits)**.

**Compound(s)**	**IC_50_/μg·ml^−1^**	**Lower limit/μg·ml^−1^**	**Upper limit/μg·ml^−1^**
Luteolin	15.642	10.459	28.044
L-Epicatechin^*^	44.247	33.025	74.599
Cianidanol	17.584	11.834	35.432
Quercetin	19.002	17.441	20.907
Luteolin & Cianidanol	11.566	5.604	24.265
Luteolin & Quercetin	14.721	10.117	23.993

* Theoretical prediction

The correlation analysis of two factors could be obtained from this model, indicating that the synergistic effects and concentrations of flavonoids were in a dose-dependent manner in a certain concentration range. In order to verify this deduction, we determined the IC_50_ value of the combination of Luteolin and Cianidanol (1:15), Luteolin and Quercetin (1:7) by microcalorimetry. The results showed that the combination of Luteolin and Cianidanol (*P*_*L*+*C*_ = 1.425), Luteolin and Quercetin (*P*_*L*+*Q*_ = 1.129) had synergetic effects, and the former couple showed stronger synergistic effect. Flavonoids had many different types of chemical structure. Luteolin and Quercetin had similar structural framework, but Quercetin had one more hydroxyl than Luteolin, thus we speculated that the antibacterial action sites of these two compounds may well be semblable. The chemical structures of Cianidanol and Luteolin are various, Luteolin has a unique carbon-carbon double bond and carbonyl compared to Cianidanol in a conjugate relationship. Compared with Luteolin, Cianidanol with a unique hydroxyl has a distinct synergistic effect on its anti-bacterial activity. The results could lay the foundation for exploring the synergistic mechanism of Luteolin and Cianidanol.

There may well exist other uncontrollable factors in UPLC with much larger peak area of substances not included as marker, thus their antimicrobial activities were not displayed under the application of quaternary quadratic general rotary unitized design.

The results of two-factor interaction effect analysis showed that Luteolin and Cianidanol had synergistic effect at high concentration. Interestingly, they were antagonistic in low concentration. Herein, a conclusion that the four flavonoids have anti-bacterial activity could be drawn. Together, this work could contribute to the quality evaluation and exploring the interaction among Luteolin, Quercetin, Cianidanol, and L-Epicatechin.

## Author contributions

YL did the writing of paper and polyamide column chromatography, Ultra High Performance Liquid Chromatography (UPLC) as well as micro-calorimetry and quaternary quadratic general rotary unitized design; YL, HX, YZ, and XX did the quaternary quadratic general rotary unitized design. MW, JW, XL, SW, KL, LW, RW, and PZ did the Ultra High Performance Liquid Chromatography (UPLC) as well as micro-calorimetry. HX, YZ, and XX supervised the project. All the authors read and approved the final manuscript.

### Conflict of interest statement

The authors declare that the research was conducted in the absence of any commercial or financial relationships that could be construed as a potential conflict of interest.

## References

[B1] Akroum S. Bendjeddou D. Satta D. Lalaoui K. (2009). Antibacterial activity and acute toxicity effect of flavonoids extracted from *Mentha longifolia*. Am. Eurasia. J. Sci. Res. 4, 93–96.

[B2] Baldoni D. Hermann H. Frei R. Trampuz A. Steinhuber A. (2009). Performance of microcalorimetry for early detection of methicillin resistance in clinical isolates of *Staphylococcus aureus*. J. Clin. Microbiol. 47, 774–776. 10.1128/JCM.02374-08 19158262 PMC2650961

[B3] Braissant O. Wirz D. Gopfert B. Daniels A. U. (2010). “The heat is on”: rapid microcalorimetric detection of mycobacteria in culture. Tuberculosis (Edinb) 90, 57–59. 10.1016/j.tube.2009.11.001 19969505

[B4] Chen J. Wang F. Liu J. Lee F. S. Wang X. Yang H. (2008). Analysis of alkaloids in *Coptis chinensis* Franch by accelerated solvent extraction combined with ultra performance liquid chromatographic analysis with photodiode array and tandem mass spectrometry detections. Anal. Chim. Acta 613, 184–195. 10.1016/j.aca.2008.02.060 18395058

[B5] Chen Y. Zhu S. B. Xie M. Y. Nie S. P. Liu W. Li C.. (2008). Quality control and original discrimination of *Ganoderma lucidum* based on high-performance liquid chromatographic fingerprints and combined chemometrics methods. Anal. Chim. Acta 623, 146–156. 10.1016/j.aca.2008.06.018 18620918

[B6] Eumkeb G. Siriwong S. Thumanu K. (2012). Synergistic activity of luteolin and amoxicillin combination against amoxicillin-resistant *Escherichia coli* and mode of action. J. Photochem. Photobiol. B 117, 247–253. 10.1016/j.jphotobiol.2012.10.006 23159507

[B7] Kabanova N. Stulova I. Vilu R. (2012). Microcalorimetric study of the growth of bacterial colonies of *Lactococcus lactis* IL1403 in agar gels. Food Microbiol. 29, 67–79. 10.1016/j.fm.2011.08.018 22029920

[B8] Khan M. R. Omoloso A. D. (2002). Antibacterial and antifungal activities of *Dracontomelon dao*. Fitoterapia 73, 327–330. 10.1016/S0367-326X(02)00076-X 12234577

[B9] Kong W. Wang X. Xing C. Jin X. Xiao X. H. Zhao Y. L.. (2011). Screening for novel antibacterial agents based on the activities of compounds on metabolism of *Escherichia coli*: a microcalorimetric study. J. Hazard. Mater. 185, 346–352. 10.1016/j.jhazmat.2010.09.040 20926184

[B10] Kong W. Zhao Y. L. Xing X. Ma X. Sun X. Yang M.. (2015). Antibacterial evaluation of flavonoid compounds against *E. coli* by microcalorimetry and chemometrics. Appl. Microbiol. Biotechnol. 99, 6049–6058. 10.1007/s00253-015-6711-1 26051672

[B11] Kong W. J. Zhao Y. L. Xiao X. H. Li Z. L. Jin C. Li H. B. (2009a). Investigation of the anti-fungal activity of coptisine on *Candida albicans* growth by microcalorimetry combined with principal component analysis. J. Appl. Microbiol. 107, 1072–1080. 10.1111/j.1365-2672.2009.04292.x 19426275

[B12] Kong W. J. Zhao Y. L. Xiao X. H. Li Z. L. Ren Y. S. (2009b). Action of palmatine on *Tetrahymena thermophila* BF5 growth investigated by microcalorimetry. J. Hazard. Mater. 168, 609–613. 10.1016/j.jhazmat.2009.02.071 19286310

[B13] Liu S. X. Zhao Y. L. Zeng N. Liu T. Zhang Y. Han B. (2013). Anti-bacterial effect of four extracts from leaves of *Dracontomelon dao* on *Escherichia coli* growth using microcalorimetry coupled with principal component analysis. J. Therm. Anal. Calorimetry 116, 491–497. 10.1007/s10973-013-3516-2

[B14] Liu T. Zhao Y. L. Wang J. B. Zhou X. Sun Z. Zheng Q. (2013). Action of crude Radix Aconiti Lateralis (Fuzi) and its processed products on splenic lymphocytes growth investigated by microcalorimetry. Therm. Acta 571, 1–7. 10.1016/j.tca.2013.07.031

[B15] Lv P. C. Li H. Q. Xue J. Y. Shi L. Zhu H. L. (2009). Synthesis and biological evaluation of novel luteolin derivatives as antibacterial agents. Eur. J. Med. Chem. 44, 908–914. 10.1016/j.ejmech.2008.01.013 18313801

[B16] Manneck T. Braissant O. Haggenmuller Y. Keiser J. (2011). Isothermal microcalorimetry to study drugs against *Schistosoma mansoni*. J. Clin. Microbiol. 49, 1217–1225. 10.1128/JCM.02382-10 21270220 PMC3122815

[B17] Ren Y.-s. Yan D. Zhang P. Li H.-b. Feng X. Zhang Y.-m.. (2010). Hemagglutination activity of radix isatidis detected by microcalorimetry. Yao Xue Xue Bao 45, 1028–1034. 10.16438/j.0513-4870.2010.08.006 21351590

[B18] Sondi I. Salopek-Sondi B. (2004). Silver nanoparticles as antimicrobial agent: a case study on *E. coli* as a model for gram-negative bacteria. J. Colloid Interface Sci. 275, 177–182. 10.1016/j.jcis.2004.02.012 15158396

[B19] Su X. F. Liang Z. Y. Zhang Y. X. (2008). Study on the chemical constituents of essential oil from the skins of stem of *Dracontomelon dao* (Blanco) Merr. et Rolfe. Lishizhen Med. Mat. Med. Res. 19, 1640–1641.

[B20] von A. H. Wirz U. D. Daniels A. U. (2009). Isothermal micro calorimetry: a new method for MIC determinations: results for 12 antibiotics and reference strains of *E. coli* and *S. aureus*. BMC Microbiol. 9:106. 10.1186/1471-2180-9-106 19470161 PMC2692853

[B21] Vor T. Dyckmans J. Flessa H. Beese F. (2002). Use of microcalorimetry to study microbial activity during the transition from oxic to anoxic conditions. Biol. Fertil. Soils 36, 66–71. 10.1007/s00374-002-0510-4

[B22] Wang F. Yao J. Chen H. Chen K. Trebse P. Zaray G. (2010). Comparative toxicity of chlorpyrifos and its oxon derivatives to soil microbial activity by combined methods. Chemosphere 78, 319–326. 10.1016/j.chemosphere.2009.10.030 19900695

[B23] Wenzler T. Steinhuber A. Wittlin S. Scheurer C. Brun R. Trampuz A. (2012). Isothermal microcalorimetry, a new tool to monitor drug action against *Trypanosoma brucei* and *Plasmodium falciparum*. PLoS. Negl. Trop. Dis. 6:e1668. 10.1371/journal.pntd.0001668 22679520 PMC3367992

[B24] Wu M. Q. Qu F. Zhao Y. L. Wang J. B. Su H. Chen C. (2015). Microcalorimetry and turbidimetry to investigate the anti-bacterial activities of five fractions from the leaves of *Dracontomelon dao* on P. aeruginosa. J. Therm. Anal. Calorimetry 123, 2367–2376. 10.1007/s10973-015-4932-2

[B25] Xie C. L. Tang H. K. Song Z. H. Qu S. S. Liao Y. T. Liu H. S. (1988). Microcalorimetric study of bacterial growth. Therm. Acta 123, 33–41. 10.1016/0040-6031(88)80007-8

[B26] Yi L. Z. Yuan D. L. Liang Y. Z. Xie P. S. Zhao Y. L. (2007). Quality control and discrimination of pericarpium citri reticulatae and pericarpium citri reticulatae viride based on high-performance liquid chromatographic fingerprints and multivariate statistical analysis. Anal. Chim. Acta 588, 207–215. 10.1016/j.aca.2007.02.012 17386812

[B27] Yi Z. B. Yan Y. Liang Y. Z. Bao Z. (2007). Evaluation of the antimicrobial mode of berberine by LC/ESI-MS combined with principal component analysis. J. Pharm. Biomed. Anal. 44, 301–304. 10.1016/j.jpba.2007.02.018 17383137

[B28] Zhao Y. L. Liu S. X. Qu F. Wang J. B. Hu Y. Zhang P. (2015). Microcalorimetry coupled with principal component analysis for investigating the anti-*Staphylococcus aureus* effects of different extracted fractions from *Dracontomelon dao*. J. Therm Anal. Calorimetry 120, 913–920. 10.1007/s10973-014-4268-3

[B29] Zhao Y. L. Wang J. B. Zhang P. Shan L. M. Li R. S. Xiao X. H. (2010). Microcalorimetric study of the opposing effects of ginsenosides Rg1 and Rb1 on the growth of mice splenic lymphocytes. J. Therm. Anal. Calorimetry 104, 357–363. 10.1007/s10973-010-1003-6

[B30] Zhao Y. L. Wei S. Z. Wang J. B. Zhang P. Li R. Xiao X. H. (2011). Microcalorimetry coupled with principal component analysis for comparing the effects of two Panax species on mice splenic lymphocytes. J. Therm. Anal. Calorimetry 111, 1669–1674. 10.1007/s10973-011-2060-1

[B31] Zheng Q. Zhao Y. L. Wang J. B. Liu T. Zhang B. Gong M.. (2014). Spectrum-effect relationships between UPLC fingerprints and bioactivities of crude secondary roots of Aconitum carmichaelii Debeaux (Fuzi) and its three processed products on mitochondrial growth coupled with canonical correlation analysis. J. Ethnopharmacol. 153, 615–623. 10.1016/j.jep.2014.03.011 24632114

